# Permeability of Normal Mucous Membrane

**Published:** 1916-09

**Authors:** P. R. Stillman


### THE PERMEABILITY OF NORMAL MUCOUS MEMBRANE BY BACTERIA.

---

The reports of Hilgermann's and Flicker's experiments on animals regarding the permeability of bacteria into the normal mucous membrane seem to throw some light upon the subject.

Hilgermann found that when young rabbits and guinea-pigs were fed with tubercle bacilli, these organisms did pass through all portions of the digestive tract epithelium, but more especially the upper part. This passage was not due to lesions of the integument, nor was there evidence of penetration in consequence of an irritation produced by colonies acting locally. The contrary was observed in the normal adult animal, except when the vitality was lowered by extreme hunger and fatigue. He concluded that the mucous membrane, under certain conditions, was lacking in the natural protective element which normally inhibits penetration. Flicker's experiments show that these conditions lend susceptibility to penetration, and that the best nourished and strongest organisms are highly sensitive to infection when subjected to these conditions. He induced hunger and, by the use of a treadmill, fatigue in dogs, and observed that its effects facilitated infection. He thought it probable that, in the time of greatly increased muscular exercise, the body cells generally suffer a temporary check in their metabolism and in their ability to liberate energy. This infirmity in some way influences the epithelium so as to favor the passage of bacteria. I am satisfied that these references partly explain the infection in the case I have described.

### BACTERIAL BALANCE.

The alimentary tract from the nasal meatus and the lips to its distant vent has a continuous unbroken lining of mucous membrane, and within its confines it harbors countless millions of micro-organic life. Herter estimates the number of both dead and living organisms in the daily excreta of the average normal, healthy human, at 126,000,000,000.

Bassler estimates the average dry-stool specimen of the healthy individual to be composed of from one-quarter to one-third in bulk of solid bacteria. It is not possible for us to comprehend the infinity of these bacterial units. These facts are cited in order to emphasize the relationship these figures bear to the bacteriology of this subject. While food and drink, as carriers, doubtless account for part of the bulk, it is certainly true that there are constantly incubating in a pathological mouth organisms which are not benign in their action upon the system. To what extent this oral flora contributes to the whole we are not prepared to say; but it may be safely assumed that a certain proportion has been incubated in the mouth.

The laws which govern bacterial life are almost identical with those which govern biological life, one variety depending upon another for food and subsistence. No one variety is dangerous until it has increased numerically and the bacterial life is thrown out of balance, causing a war within the confines of our body.

### THE DENTIST'S DUTIES OF PREVENTION.

When we realize that organisms of low pathogenicity commonly found in the mouth may readily and rapidly develop a high degree of pathogenic virility through an alteration of environment, we must recognize our responsibility in the matter of oral prophylaxis and the treatment of periodontic disease. Directly in the line of vision of the dental eye appear, first, the indications of disturbances in body metabolism. We observe a reddening of the border of the gums with the always accompanying hypostasis of the vessels; next we note copious deposits of salivary calculus, or the blue spot in the septal tissue denoting deposits of another type; again a pus sinus for a chronic apical abscess. Pyorrhea we see from its initial attack upon the border of the gum to the deformity of the advanced lesion.

Let no dentist despise the work that lies here at hand, nor underestimate the import of these significant symptoms, for these are indices of incipient disease, and here are spread before our eyes, signs which reveal disturbance in the metabolic process, so evident as to be unmistakable, offering premonitory evidence of maladies to follow at a time when symptoms are recognizable nowhere else in the body.—P. R. Stillman, in August *Cosmos*.

